# The History of Armand Trousseau and Cancer-Associated Thrombosis

**DOI:** 10.3390/cancers11020158

**Published:** 2019-01-31

**Authors:** Pat Metharom, Marco Falasca, Michael C Berndt

**Affiliations:** 1 Platelet Research Laboratory, Curtin Health Innovation Research Institute, Curtin University, Perth 6100, Australia; 2 Metabolic Signalling Group, School of Biomedical Sciences, Curtin Health Innovation Research Institute, Curtin University, Perth 6102, Australia; m.falasca@curtin.edu.au; 3 School of Medicine, Curtin University, Perth 6100, Australia; m.berndt@curtin.edu.au

*“Je suis perdu; une phlegmatia qui vient de se déclarer cette nuit, ne me laisse aucun doute sur nature de mon mal.”*

—Armand Trousseau [1]

“I am lost; a phlebitis which has declared itself this night leaves me no doubt about the nature of my illness.”

Our history is littered with tales of great physicians and scientists succumbing to diseases associated with their field of endeavour. Marie Curie died from pernicious aplastic anaemia caused by her long-term research with radioactive materials [2]. René Laennec perished from tuberculosis, a disease to which he devoted much of his time and, as legend would have it, diagnosed by his nephew using the stethoscope Laennec himself invented [3]. With the tragedy of heart-breaking self-diagnosis, Armand Trousseau (1801–1867), another celebrated French citizen of his time, also had his name firmly ingrained in medical history, indelibly linked to the field of cancer and thrombosis. An eloquent and popular orator and practitioner, Trousseau was renowned for his wit, observational skills and accurate diagnosis of various ailments [4]. Trained under the guidance of the highly respected physician Pierre Bretonneau, Trousseau made his name working on yellow fever and laryngeal diseases and popularised the use of tracheotomy for the treatment of croup and the management of diphtheria [5]. His name is associated with several medical diagnoses; Trousseau’s point, Trousseau’s sign, Trousseau’s spot and Trousseau’s syndrome [6], but it is the last eponym that Trousseau is most renowned for. Also known as Trousseau’s sign of malignancy, Trousseau’s syndrome describes a spontaneous migratory blood clot associated with cancer. 

In the chapter entitled *“Phlegmatia Alba Dolens”*, originally published in French in 1865 [7], from his most famous work, the compilation of his delivered lectures, *Clinique Medicale de l’Hotel-Dieu de Paris,* Trousseau discussed several detailed cases and put forth the symptom of migratory thrombophlebitis as a valuable diagnostic element of visceral cancer.

“I have long been struck with the frequency with which cancerous patients are affected with painful oedema in the superior or inferior extremities, whether one or other was the seat of cancer. This frequent concurrence of phlegmasia alba dolens with an appreciable cancerous tumor led me to the inquiry whether a relationship of cause and effect did not exist between the two, and whether the phlegmasia was not the consequence of the cancerous cachexia. I have since that period had an opportunity of observing other cases of painful oedema, in which, at the autopsy, I found visceral cancer, but in which during life, there was no appreciable cancerous tumor; and in which there existed a cachexia referable neither to the tubercular diathesis, the puerperal state, nor chlorosis. I have thus been led to the conclusion that when there is a cachectic state not attributable to the tuberculous diathesis nor to the puerperal state, there is most probably a cancerous tumor in some organ.” [8]. 

He also astutely observed, quite ahead of his time, that a “*particular condition*” in the blood, speculated to be “*excess of fibrin, and an increase of white globules*”, was the primary cause of thrombosis and that the change (hypercoagulable state) was also evident in many other disorders [8]. Although the first case of blood clot in cancer was noted by Jean-Baptiste Bouillaud in a publication several decades earlier [9], Bouillaud’s contribution to the field was limited, since he preferred the studies of cardiology and neurology [10]. Nevertheless, the keen observation of Bouillaud was acknowledged by Trousseau in his seminal lecture to be the basis of all venous obstruction research made in France since 1823 [8].

Trousseau retired from his prestigious post at the Faculty of Medicine and the Hôtel-Dieu de Paris (the oldest hospital in Paris) in the summer of 1866. He was tired and suffering from ill health and, as months progressed, he began to analyse his symptoms; weight loss, lack of appetite, repeated haemorrhages and stomach pains that were becoming increasingly resistant to opium [1]. For a while, he found no palpable tumour and, most importantly, no painful inflammation or oedema along the venous paths. But once he detected the missing sign he had searched for in so many of his patients, his diagnosis of gastric cancer was confirmed, and he lived for several more months in agony for its discovery, for he knew better than anyone the fatal outcome of his illness (Figure 1). 

A hundred and fifty years have passed since Trousseau’s death, and we have since learned more about the underlying mechanisms involved in cancer-associated thrombosis. For instance, platelets, which were not clearly identified as a distinct cellular entity until 1882 [12], are now recognized to have a key regulatory role in thrombus formation as well as an appreciable function in promoting tumour metastasis. The advent of new technologies in genomics and proteomics have also allowed us to understand better the molecular basis of cancer. 

This Special Issue will highlight and summarise the background of the field as well as recent research studies and technical papers. In this Editorial, we summarize the main themes discussed in this issue and highlight the relevance of some of the findings. Abdol Razak et al. give an introductory overview of the main thrombotic complication in cancer patients focussing on multiple mechanisms involved in cancer-associated venous thromboembolism (VTE) [13]. Frere et al. contribute a summary of the main recent advances in the prevention and treatment of cancer-associated VTE and the utilization of personalized risk factors for patient stratification for VTE risk [14]. A specific focus is given by Scotté et al. to the tailored management of cancer-associated thrombosis in frail patients, particularly those of advanced age and comorbidities [15]. Analogously, Zanetto et al. review the current knowledge on thrombotic complications in patients with liver cirrhosis and hepatocellular carcinoma (HCC) [16]. Their conclusion is that the identification of cirrhotic patients with HCC with the highest prothrombotic profile would provide the rationale for personalized thromboprophylaxis. Riondino et al. give an overview of candidate biomarkers and prediction models at present under scrutiny to be used for risk prediction of chemotherapy-associated VTE [17]. Alexander et al. propose a novel thromboembolism prediction model to be used for targeted thromboprophylaxis in Non-Small-Cell Lung Cancer [18]. They are currently validating this model in a multicentre randomised interventional study. Al-Samkary and Connors provide a comprehensive review on the use of direct oral anticoagulants in the treatment of VTE, with particular emphasis on efficacy and safety in cancer patients [19]. Bruno et al. discuss the mechanism by which platelets can promote cancer, with a particular focus on metastasis [20]. They note that the active role played by platelets in cancer provides the rationale for the potential use of antithrombotic agents for both the prevention of cancer and the lowering of metastatic spread and consequent mortality. Specific attention is assigned to the promising efficacy of aspirin and clopidogrel. Similarly, Grandoni and Alberio review published and ongoing studies on the direct oral anticoagulant (DOACs) use in cancer patients [21]. The existence of a cross-talk between platelets and cancer cells is extensively reviewed by Plantureux et al. [22]. Indeed, cancer cells can influence both platelet status and function. The generation of tumour-educated platelets provides a novel therapeutic strategy for cancer-associated thrombosis and cancer progression. Reddel et al. focus on the role played by thrombin in cancer–platelet cross-talk, with consequent increases in blood coagulation and cancer progression and metastasis [23]. Wojtukiewicz et al. address the functional significance of Endothelial Protein C Receptor (EPCR), Protease-Activated Receptor-1 (PAR-1) and their interplay in cancer growth and metastatic spread [24]. Finally, Luu et al. focus on bone marrow dysfunction and how this influences platelet function [25]. 

It has been truly a delight assimilating this collection of manuscripts regarding the state of the art of the role of thrombosis and haemostasis in cancer. While we are well aware that this is not a fully comprehensive overview of the field, we hope that this represents for the reader a valuable source of background knowledge and latest advances. We believe that this is an area in rapid expansion, and future studies will undoubtedly bring novel exciting findings for the prevention and treatment of cancer-associated thrombosis.

## Figures and Tables

**Figure 1:**
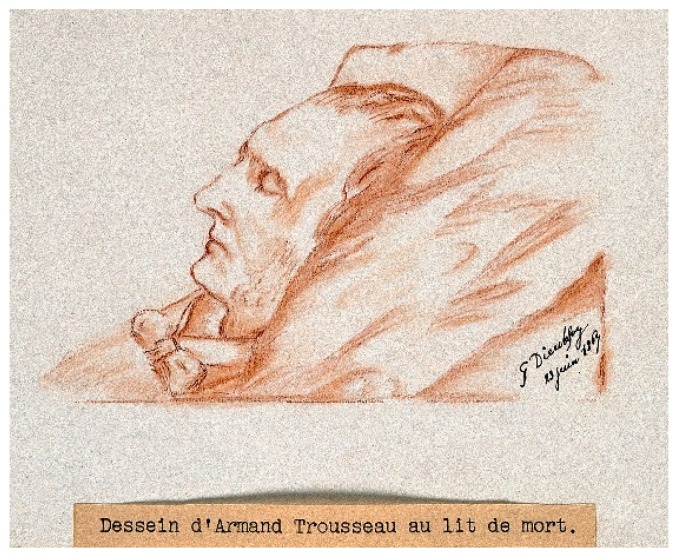
A chalk drawing of Trousseau on his deathbed by one of his students, Dieulafoy G. [11].
